# The Early Time Course of Compensatory Face Processing in Congenital Prosopagnosia

**DOI:** 10.1371/journal.pone.0011482

**Published:** 2010-07-21

**Authors:** Rainer Stollhoff, Jürgen Jost, Tobias Elze, Ingo Kennerknecht

**Affiliations:** 1 Max Planck Institute for Mathematics in the Sciences, Leipzig, Germany; 2 Santa Fe Institute, Santa Fe, New Mexico, United States of America; 3 Institute of Human Genetics, Westfälische Wilhelms-Universität, Münster, Germany; National Institute of Mental Health, United States of America

## Abstract

**Background:**

Prosopagnosia is a selective deficit in facial identification which can be either acquired, (e.g., after brain damage), or present from birth (congenital). The face recognition deficit in prosopagnosia is characterized by worse accuracy, longer reaction times, more dispersed gaze behavior and a strong reliance on featural processing.

**Methods/Principal Findings:**

We introduce a conceptual model of an apperceptive/associative type of congenital prosopagnosia where a deficit in holistic processing is compensated by a serial inspection of isolated, informative features. Based on the model proposed we investigated performance differences in different face and shoe identification tasks between a group of 16 participants with congenital prosopagnosia and a group of 36 age-matched controls. Given enough training and unlimited stimulus presentation prosopagnosics achieved normal face identification accuracy evincing longer reaction times. The latter increase was paralleled by an equally-sized increase in stimulus presentation times needed achieve an accuracy of 80%. When the inspection time of stimuli was limited (50ms to 750ms), prosopagnosics only showed worse accuracy but no difference in reaction time. Tested for the ability to generalize from frontal to rotated views, prosopagnosics performed worse than controls across all rotation angles but the magnitude of the deficit didn't change with increasing rotation. All group differences in accuracy, reaction or presentation times were selective to face stimuli and didn't extend to shoes.

**Conclusions/Significance:**

Our study provides a characterization of congenital prosopagnosia in terms of early processing differences. More specifically, compensatory processing in congenital prosopagnosia requires an inspection of faces that is sufficiently long to allow for sequential focusing on informative features. This characterization of dysfunctional processing in prosopagnosia further emphasizes fast and holistic information encoding as two defining characteristics of normal face processing.

## Introduction

Prosopagnosia, colloquially also referred to as “face-blindness”, was first defined by Bodamer as a selective deficit in the specific task of face identification [1], although the deficit has been reported previously in conjunction with more general object recognition deficits [2]–[4]. Since then, prosopagnosia has mostly been observed in cases of acquired prosopagnosia, where the deficit was caused by neurological damage following e.g. intoxication, head injury or encephalopathy [1], [5]–[9].

Recently, more and more cases of prosopagnosia have been reported where the deficit was not acquired due to an accident, but presumably present from birth, i.e. congenital [9]–[17]. In contrast to the rare acquired form, the congenital form is among the most common anomalies in humans with a prevalence of 2.5% and is almost always hereditary [16]–[20]. Notwithstanding ongoing discussions on the nature of this congenital form, here we will continue to refer to all cases of prosopagnosia without any exogenous cause as cases of congenital prosopagnosia (CP), without explicitly addressing the question of heritability or developmental influences.

The face recognition deficit in CP can be as profound as in the acquired form [9] and equally selective such that only facial identification is impaired while all other aspects of face and object recognition remain intact [21]. However, the selectivity of the deficit is questioned by recent reports on deficits in the processing of biological motion [22] and decreases in the subjective vividness of visual mental imagery [23]. Overall, cases of CP often display heterogeneous symptoms [24] which has so far prevented a stringent categorization of congenital prosopagnosia according to phenotypical symptoms.

In this study, we investigate the face recognition deficit in CP in relation to the following qualitative model of facial information processing. We propose that normally for identification faces are encoded incrementally by holistic processing [25] of informative snapshots of faces. The model is similar to existing models of face perception [26] but focuses on a more detailed description of the process of structural encoding. However, our understanding of structural encoding is based mostly on methods of machine learning or computational vision. For example, we assume that any given snapshot always encodes a holistic representation of the information contained in all face parts. Changes of fixations between snapshots only restrict the resolution with which individual face parts can be processed but don't imply an exclusive processing of the face part fixated on, as would be the case in featural processing. Only in this sense, is the difference between featural and holistic processing a qualitative one. More generally, the difference is rather quantitative in the amount of information which is extracted and integrated for any given snapshot.

We propose that the informativeness of individual snapshots depends mainly on two factors: feature variability across members of a population and limitations in the retinal and cortical resolution of image parts. Optimally, informative regions are processed earlier on and are fixated on more often, i.e. encoded with a higher precision, than non-informative ones. The informativeness of regions is learned over repeated exposures to faces and depends more on the population exposed to and only to a lesser degree on the actual face stimulus encoded. Our assumptions are compatible with psychological studies showing the recruitment of holistic processing strategies in face recognition [25], stereotypical fixation patterns that depend neither on the fixation sequences actually employed during encoding [27], nor on the individual face perceived [28]. Although initially violating optimal information processing of individual faces, stereotypical fixation patterns can minimize difficulties in comparing translated snapshots (cf. decreases in the performance of appearance based methods for automated face recognition [29]). Fixation patterns depend on the cultural background of the observer but are independent of that of the stimulus [30]. Whether this is due to social norms, as suggested by the authors, or due to different patterns of variability across populations (cf. “other-race-effect” [31]) is still an open question. During recognition a perceived face image is matched in parallel against stored representations, and the identity is determined according to a best match. The accuracy of the matching process is limited not only by the quality of the stimulus presented but moreover by the quality of the previously stored representation. This quality increases with the number of snapshots taken during initial encoding (i.e. increased encoding time) and the informativeness of the snapshots (e.g. fixation on informative regions, retinal and cortical resolution). The recognition of individual faces is based on informative snapshots, i.e. representations that integrate information from the full face, and thus allows a faster processing than serial matching of (local) informative features used for classification [32], [33].

In contrast to normal processing, we hypothesize that in apperceptive/associative [6] types of CP facial encoding is a mostly deliberate process of extracting (local) informative features in a series of fixations or attentional shifts. As the uniqueness of isolated features, and therefore their informational content, differs between individual faces, so does the series of fixations employed to extract the information. Indeed, face processing in CP has been characterized by a reliance on featural processing [15], [34] and more dispersed fixation patterns [35], [36]. More specifically, while controls fixate almost exclusively on the eye, nose, and mouth region, participants with CP direct a small but significant amount of fixations on external features. Furthermore, the proposed compensatory featural processing is contingent on cognitive strategies of problem solving, which is in agreement with a face-specific increase in the BOLD response in frontal areas in congenital prosopagnosia [37]. In an ideal CP observer model, during the initial encoding of the stimulus a face image is scanned for informative regions and if given sufficient time a unique, optimally informative series of fixations is developed. This doesn't necessarily imply that CPs will always be able to extract the same total amount of information, only that the fixation sequence is optimal given the restriction on featural processing. Thus, depending on the task difficulty (number of target and distractor stimuli, availability of informative features,…) CPs might perform with a normal accuracy. This is in line with studies showing that participants with CP can achieve close to normal face recognition performance in standardized tests [38], however they often show longer reaction times [15].

Underlying the distinction between the proposed models for CP and normal face processing is the difference between featural and holistic encoding. Although, as argued above, we regard this as a quantitative difference in encoding, the implications on the overall process of face recognition are of a more qualitative nature: On the one hand, the incremental refinement of a universally holistic representation capturing how an individual differs from its population. On the other hand, the iterative expansion of personalized “mental lists” by adding isolated features that are unique to this specific individual. The proposed models align with explanations of the dissociation between facial identification and intact object classification as a difference in the level of visual expertise needed [39], [40]. While successful identification of individual exemplars depends on holistic image-based representations, classification of objects can be accomplished by a comparison of image parts or features (see [41] for a review of computational approaches).

The experimental assessment of the models carried out in this study focuses on the implications of the models on temporal dynamics of face recognition and the influence of stimulus transformations. More specifically, we test the following predictions: First, if given sufficient training and inspection times, CP participants might be able to achieve the same performance as controls presumably by a serial matching of isolated features. Second, for any given fixed inspection time, on average CP participants will extract less information and thereby perform worse. Third, for controls, limiting inspection time during initial encoding has a stronger influence than limiting inspection time during recognition. An interruption of the incremental refinement of a holistic representation is more detrimental than a shortened period available for holistic matching. Fourth, we hypothesized that in both cases processing relies mostly on appearance-based (i.e. pictorial) information that doesn't generalize well across rotation in depth [42]. Thus, the deficit in CP is not related to a dysfunctional generalization and on average the influence of stimulus transformation shouldn't differ between control and CP participants. In addition to testing these predictions on differences in facial identification, we investigated differences in the ability to identify novel stimuli (Nike™ sneakers resp.) in which neither group had any prior expertise and thus couldn't engage in holistic processing. This served to ascertain that possible differences in face recognition between controls and CPs can not be explained by a general decrease in visual proficiency among the CP participants.

In order to test the predictions of the proposed models and better characterize the behavioral symptoms in congenital prosopagnosia, we conducted a series of experiments, each testing different aspects of face and object recognition, with a total of 16 CP and 32 control participants. The setup of the experiments closely parallels those of other tests (e.g. Cambridge Face Recognition Test [43]), and was restricted to setups which have a direct analogue in real-life situations avoiding unrealistic conditions, e.g. scrambling or inverting a face.

In the first two experiments, a standard setting was used to test recognition of frontal images of faces (experiment 1) and shoes (experiment 2). Participants were familiarized with four individual target stimuli and later on had to identify the targets amongst a group of distractor stimuli in a two-alternative forced choice paradigm (target vs. non-target). We specifically investigated whether longer reaction times can be attributed to longer inspection of the images or a longer decisional component. First, we measured participants' reaction times under the condition of unlimited presentation. Second, we used an adaptive sampling strategy to estimate the presentation time at which a participant performs with an accuracy of 80%. Third, we contrasted individuals' reaction times with their 80%-correct presentation times. Previous studies of CP-control differences in reaction times in facial identification tasks have provided mixed results: An increase in CPs reaction times in a matching task with unlimited exposure duration [15], but no difference in reaction times in a delayed recognition task with a limited presentation time (200ms) during learning [44]. Here, participants had unlimited presentation time during learning of the stimuli. Reaction times were measured under unlimited presentation and compared to presentation times needed for equal performance. The latter hasn't been studied so far, and in itself as by comparison with reaction times provides a direct measure of possible speed-accuracy trade-offs in CP which have been proposed previously [15]. Moreover, using the same experimental design with both face and shoe stimuli allows to clarify whether possible speed-accuracy trade-offs are restricted to the processing of faces.

In experiment 3 (faces) and experiment 4 (shoes) we investigated each participants' ability to generalize stimulus recognition across rotation in depth. While recognition of stimuli taken under identical viewing conditions can be solved by image matching, rotation in depth which occurs frequently under natural viewing conditions at least diminishes the applicability of similar compensatory strategies. Previously, it was shown that normally for faces learned in a frontal view, recognition performance decreases monotonically when tested with images rotated around the vertical axis [42]. Here, we assessed whether participants with CP show a similar or more pronounced decrease in their performance to discriminate between the previously learned targets and distractors. Thus we directly investigate participants ability to generalize from a learned view (front) to a novel view of the that was never experienced before in a delayed recognition tasks.This differs from previous studies employing rotated images in a matching task where target and samples where always shown in the same view [15], differing in illumination [45], or studies with a delayed recognition tasks where stimuli were learned in every viewing condition prior to being tested. In order to isolate the influence of rotation and avoid statistical ceiling (or bottom) effects in the performance, images were displayed for different durations estimated according to individual performance in experiments 1 and 2 respectively.

The setup of experiments 1 and 2 entailed the presentation of stimuli for different durations that were individually determined for each CP participant and their respective matched controls. In experiments 5 and 6, four fixed presentation times were used, identical for all participants and chosen to separate between the times needed for preparation and execution of one or multiple saccades. In addition, we investigated whether there are differences in the effect of tachystocopic presentation depending on whether they are applied during the encoding, i.e. learning, of a novel face (experiment 5) or during the decoding, i.e. recognition, of a previously learned face (experiment 6). Previous studies of patients with acquired prosopagnosia have shown a more pronounced deficit after limiting exposure to “tachystocopic” presentation [46] - anecdotal evidence only, or to presentations of 1500 or 5000ms [40]. In a study of CP face recognition employing a delayed recognition task [44], presentation time was limited during learning (200ms) but unlimited during recognition, similar to our experiment 5 but without any variation of the limited presentation duration.

Differences in the age of participants as well as observations of cognitive heterogeneity in CP participants [24] prompted us to resort to a threefold statistical analysis: First we tested for group differences in location using a robust, non-parametric Wilcoxon rank-sum test on the raw data (average values - mean - for each participant). Second, in order to account for inter-individual differences in age and to test for group differences in the influence of experimental parameters on participants' performance, we used generalised linear mixed models (GLMMs, see [47] for a review). Third, based on fitted GLMMs, for each participant we calculated individual residuals as the difference between actual outcomes and the outcome that would be expected based on average control performance. In this sense, residuals capture individual deviations from a hypothetical, average control, thereby providing a straightforward abnormality score to measure the size of individual CP participants' deficit.

## Results

### Experiments 1 and 2

For the face stimuli used in experiment 1, CP participants made more mistakes than controls during the initial feedback training. Also, reaction times - measured during subsequent feedback trainings - as well as the presentation times individual participants needed to achieve 80% correct recognition rates were larger among CP participants. Difference in reaction times are of the same magnitude as difference in presentation times, which suggest that increased reaction times for CP participants are due to a longer inspection of the stimulus as opposed to a longer time to reach a decision. For the shoe stimuli used in experiment 2, control and CP participants' performance, reaction and presentation times didn't differ significantly.

#### Performance during feedback learning

On average participants with congenital prosopagnosia needed more training than controls. During the initial training prior to the first testing they made more mistakes than controls in the face recognition test (means 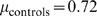, and 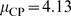; Wilcoxon rank sum test, 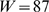, 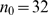, 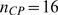, 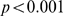 two-sided), but not in the shoe recognition test (means 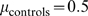, 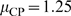; 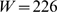, 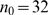, 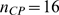, 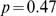 two-sided). For controls, both tasks were of comparable difficulty (Wilcoxon signed rank test, 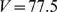, 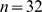, 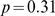 two-sided)

#### Reaction times during feedback training

**Figure 1:**
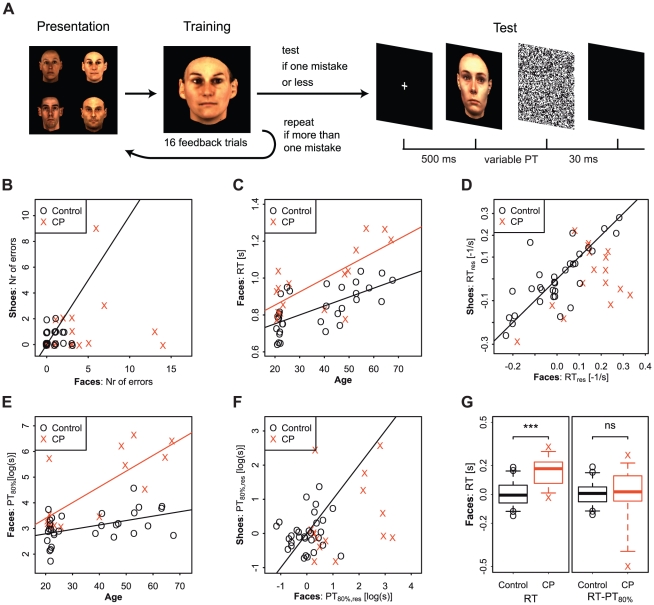
Contrasting face and shoe recognition for frontal images. (**A**) In experiments 1–4 participants were first presented with 4 target stimuli, and were trained during at least 16 feedback trials prior to the test. During feedback training, participants with congenital prosopagnosia (CP) on average made more mistakes during initial learning than controls only for faces but not for shoes (**B**, solid line for equality). Reaction times during later training trials with unlimited viewing were strongly influenced by participants' age (**C**, shown for faces only, solid lines represent linear model fits). Comparison of residuals, which account for age related differences, revealed longer reaction times for CPs compared to controls for face stimuli but not for shoe stimuli (**D**, RT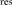, inverse transformation i.e. 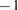/RT see Methods). In face and shoe recognition, the presentation time needed to achieve 80% correct recognition (PT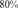) increases with age (**E** shown for faces only, solid lines represent linear model fits). CP participants needed longer presentation times than controls in tests for face recognition (Exp. 1) but not for shoe recognition (Exp. 2). (**F** comparing residuals PT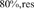). Group differences in mean reaction time for faces stimuli (**G** left boxplots, RTs centered around control mean) vanished after subtracting PT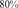 presentation time ((**G** right boxplots, values centered around control mean): CP participants needed to inspect face stimuli longer than controls. Boxplot shows group distributions (whiskers: 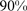 CI); significance values according to a Wilcoxon rank sum test.

Analysis of raw data, after outlier removal, revealed that on average CP participants had longer mean reaction times than controls for faces (means 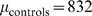 ms, 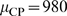 ms; Wilcoxon rank sum, 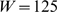, 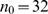, 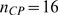, 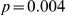 two-sided), but not for shoes (means 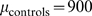
ms, 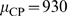
ms; 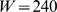, 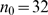, 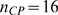, 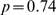 two-sided). For controls, mean reaction times were faster for face than for shoe recognition (Wilcoxon signed rank test, 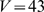, 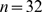, 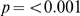 two-sided). In both tasks reaction times increased with age (see Figure 1 C for faces).

After accounting for age related changes in reaction time, differences between the groups are still significant for faces (LR-test of main effect, 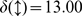, 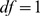, 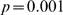, 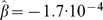, 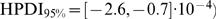), but not for shoes (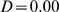, 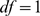, 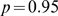). In contrast to group differences in the mean, in both cases the influence of training block number on reaction time didn't differ between groups (LR-test of first-order against main effect model, 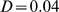, 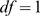, 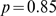 for faces, and 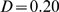, 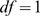, 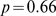 for shoes). Thus, in both groups training led to a comparable decrease in the reaction times.

A comparison of residuals revealed that participants with CP had longer reaction times than expected (larger residuals of −1/(reaction time)) for faces ((Wilcoxon rank sum, 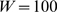, 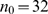, 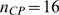, 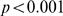 one-sided) but not for shoes (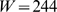, 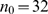, 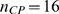, 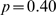 one-sided). The increase in reaction time was selective for faces for most CP participants: 13 out of 16 CP participants had higher residuals in the face task compared to the shoe task (see Figure 1 D).

#### Presentation times

All control participants only needed very short presentation times to perform at an 80% correct level in both the face (individual PT
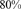
s range between 6 ms and 47 ms) and shoe recognition task (between 9 ms and 102 ms) which is well below the time needed for controlled eye movements (i.e. less than 200 ms). In contrast, several participants with congenital prosopagnosia required far longer presentation times to accurately recognize faces (21 ms to 766 ms) and/or shoes (7 ms to 462 ms). In both groups age had a strong confounding influence on the presentation time needed (shown for faces in Figure 1 E)

Comparing both groups, PT
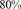
s were larger for CP than control participants for faces (medians of 34.2 ms and 20.9 ms respectively; Wilcoxon rank sum test, 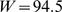, 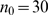, 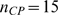, 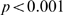 one-sided) but not for shoes (medians of 20 ms and 17.3 ms respectively; 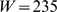, 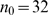, 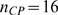, 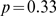 one-sided). Analogously, using model based comparisons revealed a significant group difference in the log-transformed PT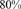 for faces (LR-test, 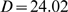, 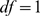, 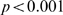) but not for shoes (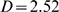, 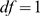, 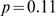).

Compared to the control group CP participants had larger residuals in the log-transformed PT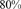 - needed longer presentation times than expected - for the face task (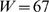, 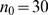, 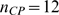, 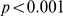 one-sided) but not for the shoe task (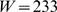, 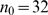, 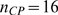, 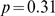 one-sided).

#### Comparison of reaction times and presentation times

While CP participants had significantly longer mean reaction times recognizing faces in experiment 2 (see above), there is no significant group difference left after we subtracted the time participants' needed to perform at an 80% correct level (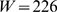, 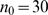, 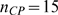, 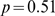) and thereby accounted for differences in the time participants need to inspect a stimulus (see Figure 1 G). Thus, while CP participants needed to inspect face images longer than controls, the time taken to make a decision didn't seem to differ.

### Experiments 3 and 4

CP participants made more mistakes in recognizing rotated face images than controls. However, the difference in performance did not change across the rotation angles tested. For shoe stimuli, no significant group difference were observed.

**Figure 2:**
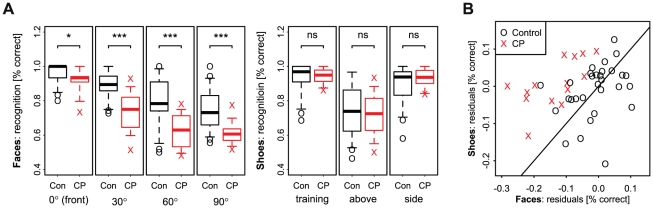
Contrasting face and shoe recognition for rotated images. In the recognition of rotated images, CP participants performed worse than controls for for faces (Exp. 3) but not for shoes (Exp. 4). This was tested for every rotation angle separately (**A** with significance values according to a Wilcoxon rank sum test). A comparison of individual residuals, which account for differences in the experimental setup, revealed that the deficit is selective for all CPs (**B**). Note that the magnitude of group differences observed for face recognition didn't change with rotation angle (Likelihood-ratio test, 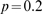).

CPs performed significantly worse than controls for faces across all rotation angles (Wilcoxon rank sum tests, 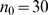, 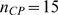, all 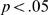 one-sided) but there was no significant difference for shoes in any rotation condition (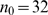, 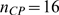, all 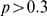 one-sided), see Figure 2 A.

Model based comparisons revealed a significant main effect for faces (LR-test of main effect, 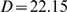, 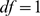, 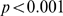), but not for shoes (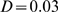, 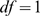, 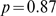). In contrast, for both types of stimuli the influence of presentation times on recognition differed between the two groups (faces: LR-test of PT interaction model against main effect model, 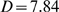, 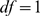, 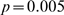; shoes: PT interaction model against nullmodel 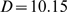, 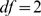, 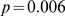). This difference was to be expected and can be attributed to the experimental setup: For CP participants, the presentation times were individualized based on the participants performance in recognizing frontal images; for control participants the assignment of presentation times was independent of individual performance. The individualized presentation times for frontal face images appear adequate to capture individual variability among CP participant also for rotated images: While control participants with longer presentation times performed better (faces: 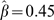, 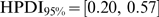; shoes: 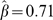, 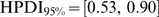), there was no effect of presentation time on performance for CP participants in recognizing faces (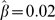, 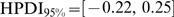) and only a slight effect in recognizing shoes (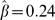, 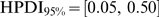). The influence of rotation on recognition performance didn't differ between CP and control participants neither for faces (LR-test of full interaction model against PT interaction model; 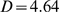, 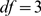, 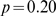) nor shoes (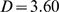, 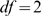, 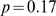).

Comparing residuals, CP participants performed worse in the recognition of rotated images for faces (Wilcoxon rank sum, 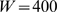, 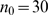, 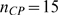, 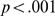 one-sided) but not for shoes (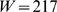, 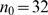, 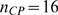, 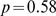 one-sided). The difference in performance between face and shoe recognition was selective for all CP participants (see figure 2 B).

### Experiment 5

In experiment 5 we tested face recognition for stimuli with a limited presentation time during initial encoding. Compared to controls, CP participants made more mistakes in recognizing target faces. Interestingly, this difference in performance was already present for presentations of only 50 ms and didn't change across different presentation times. In contrast to previous results that controls responded faster than CP participants if presentation time is unlimited, under limited presentation there was no difference in reaction times. However, while controls responded faster if the target has been previously presented longer, no such relation could be observed among CP participants.

**Figure 3:**
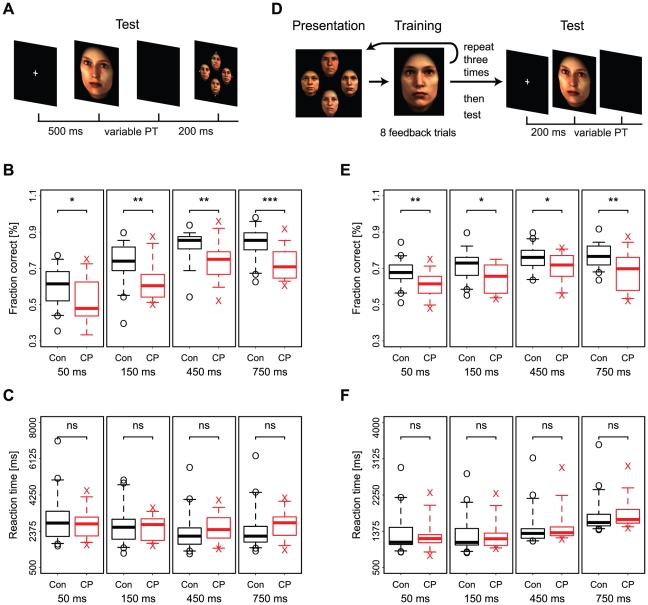
Face recognition under the constraint of limited presentation time. (**A**) In experiment 5, participants were shortly presented with a target stimulus for 50ms, 150ms, 450ms or 750ms, and after a short blank had to recognize the target in a display of four face images. For all presentation times used in experiment 5, performance of CP participants differed from controls (**B**), while there was no significant difference in reaction times (**C**). However, while controls responded faster with increasing presentation time, there was no significant influence of presentation time on CP reaction times (Likelihood-ratio test, 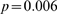). (**D**) In experiment 6, participants were repeatedly presented target faces, which - after a total of 24 feedback trials - had to be recognized in a 2-alternative forced choice paradigm. During the test, faces were presented for variable durations (50ms, 150ms, 450ms or 750ms). Independent of the duration, CP participants performed worse than controls (**E**), without significant differences in reaction times (**F**).

On average control participants performed better than CP participants for all of the presentation times tested (Wilcoxon rank sum test, all 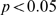, see Figure 3 B). Model based comparisons revealed a significant main effect of group (LR test of of main effect: 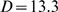, 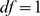, 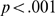). Although both groups improved in performance with increasing presentation time during the learning of the stimuli, the performance of control participants increased slightly stronger than the performance of CP participants (LR-test of full model against main effect model, 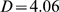, 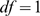, 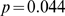). The increase in performance was lower in CP participants compared to controls (difference 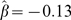, 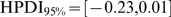). Thus, control participants profited more from increased presentation times during learning than CP participants.

There was no difference in average reaction time between control and CP participants for any of the presentation times (Wilcoxon rank sum test, all 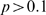 two-sided, see Figure 3 C). This was confirmed by a model comparison (LR-test of main effect: 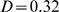, 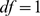, 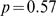). However, a significant difference in the influence of presentation time during learning on reaction time during recognition was found (LR-test of full model against null model: 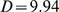, 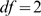, 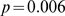): While controls participants' reaction times decreased with increasing learning time (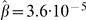, 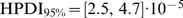, note that coefficients are with respect to an inverse scale), there was no significant influence for CP participants (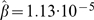, 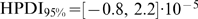, inverse scale).

Residuals with respect to the nullmodel are greater for CP participants compared to control participants in recognition performance (Wilcoxon rank sum test, 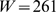, 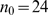, 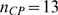, 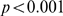 one-sided) but there's no difference in residuals of reaction times (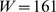, 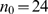, 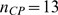, 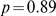 two-sided).

### Experiment 6

Experiment 6 assessed participants performance in recognizing faces that were presented for a limited duration during the recognition phase. Similar to experiment 5, CP participants made more mistakes in recognizing target faces than controls. Again, this difference in performance was already present for presentations of only 50 ms but compared to experiment 5 the difference didn't increase with increasing different presentation time. There were no differences between CP participants' and controls' reaction times.

On average control participants performed better than CP participants already after a presentation of only 50 ms (Wilcoxon rank sum test, all 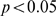, see Figure 3 E). Model based comparisons revealed a significant main effect of group (LR test of of main effect: 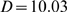, 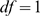, 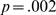). Both groups improved in performance with increasing presentation time (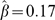, 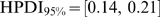), without a significant difference (LR-test of full model against main effect model, 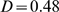, 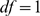, 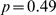).

There was no difference in average reaction time between control and CP participants for any of the presentation times (Wilcoxon rank sum test, all 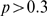 two-sided, see Figure 3 F). This was confirmed by a model comparison (LR-test of main effect: 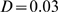, 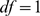, 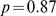). There was no significant difference in the influence of presentation time on reaction time between the groups (LR-test of full model against null model: 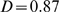, 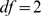, 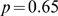).

Residuals with respect to the nullmodel are greater for CP participants compared to control participants in recognition performance (Wilcoxon rank sum test, 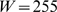, 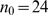, 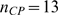, 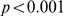 one-sided) but there's no difference in residuals of reaction times (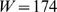, 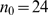, 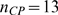, 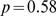 two-sided).

## Discussion

### Summary

In all of the face recognition tests there was a significant difference in performance between the group of CP participants and the control group: CP participants needed more initial training, had longer reaction times and needed longer presentation times of stimuli to achieve the same level of accuracy as compared to controls. The face recognition deficit in CP participants was present for both: frontal views of the faces, which were extensively trained, and rotated views of the faces, which were only presented as test stimuli. In contrast to the recognition of faces, there was no performance difference between the two groups in discriminating individual shoes. However, for four CP participants (HE,SE,HB,RK) the deficits seemed to extent to object identification.

Regarding our hypothesis, we first replicated findings that CP participants can achieve a face recognition accuracy comparable to controls, albeit requiring more initial training and longer reaction times. This increase in reaction time was paralleled by an equal-sized increase in the presentation time CP participants needed in order to perform at the same level as controls. Furthermore, if stimulus presentation is limited during recognition (experiment 6), these differences in reaction time during recognition vanish. Thus, CP participants can achieve normal recognition accuracy but they need to inspect the stimulus longer.

Second, CP participants showed worse recognition accuracy than controls if presentation time - and therefore the process of information extraction - was limited (experiments 5 and 6). The difference was already present for presentation times of 50ms. Thus, performance differences are not merely a function of slower processing but of differences in the processing that are present from the very beginning.

Third, for both groups limits on presentation time had a detrimental influence on recognition accuracy both if the limitation took place only during the initial encoding, learning (experiment 5), or only during matching of faces, recognition (experiment 6). It seems that the positive influence of increasing presentation times (e.g. from 50ms to 750) was more pronounced in the former case of restricted initial encoding and unlimited recognition time (see Figure 2), but a direct comparison is difficult due to the differences in experimental design. Only in experiment 5 did we observe group differences in the positive influence of prolonged presentation times on accuracy. Furthermore, this positive influence on accuracy was paralleled by a decrease in reaction times, but only among controls and not among CP participants. Thus for controls restricting inspection time during the initial encoding influences recognition accuracy and reaction time to a larger degree than for CP participants. In contrast, the difference in accuracy between CPs and controls doesn't change after increasing limited presentation times during recognition (experiment 6), where reaction times don't differ at all between both groups. Moreover, while control reaction times during recognition decreased with longer presentation times in experiment 5 they increased in experiment 6. In both experiments the stimuli were presented without masks, and it is possible that processing of the stimuli continued after their physical disappearance. But, as the results from experiment 1 indicate, the duration of post-presentation processing doesn't seem to differ between controls and CPs, at least during the recognition process.

Fourth, performance in face recognition decreased with increasing rotation angle between learned view and testing view for both CPs and controls similarly. Although this finding supports the original model put forward, the absence of group differences in the influence of rotation might have been due to different reasons. Firstly, in the experiments participants were trained only on frontal images. However, it is questionable whether frontal images are suited to the construction of 3D models [42] and whether the construction can be based on the observation of a single image at all. Secondly, isolated features might posses a certain inherent degree of transformation invariance against experimental stimulus manipulations. For example, one CP participant used cues with a high degree of rotation invariance as part of their compensatory processing or feature selection strategies. In recognizing one of the target faces, FP attended a small mole placed on the left side of the neck which was visible in frontal view and for rotations to the left but hidden for rotations to the right. Accordingly FP recognized this target face whenever it was rotated to the left and failed to recognize the target whenever it was rotated to the right, irrespective of the rotation angle.

### Models of Congenital Prosopagnosia

In this study we proposed a model for an apperceptive or associative type of CP, where the deficit is due to a failure of holistic encoding. The fallback on encoding isolated features leads to a compensatory processing that relies on actively scanning a face for informative features and leads to longer inspection times. Depending on the task difficulty, this compensatory processing might give rise to a normal accuracy in facial identification tasks. Equality of performance might be achievable especially in experimental investigations which draw on a limited number of faces each presented only in a small number of images.

Central to the proposed model of CP is a shift from holistic encoding, with face regions “weighted” according to their informativeness, to a serial scanning of isolated features, which are constructed and optimized for each face individually based on the informativeness of each feature in isolation. However, it is important to note that optimality and informativeness always depend on the processing capabilities available. With regard to fixation behaviour in controls and CP this raises the question whether the locations providing optimal information for holistic encoding actually should be the same for compensatory featural encoding in CPs. It might even be possible that for holistic processing there is no “right” location: It is important to choose and maintain a fixation spot, but the exact location is based on social norms [30].

In a recent study on training face recognition in a girl with CP [36], the authors explicitly instructed the child to focus on individual, informative features to recognize a set of familiar faces. After training, they observed an increased performance in recognizing the familiar faces as well as a change in scan paths. Prior to training the child showed a dispersed gaze behaviour, but afterwards it spent more time fixating internal features for familiar as well as novel faces. However, based on the evidence provided it can not be ruled out that the change in fixation patterns is solely due to a serial checking of all learned feature lists, e.g. recapitulating taught fixation sequences in order to be able to identify a familiar face, instead of the generalization of a fixation strategy to novel faces as proposed by the authors.

The model of an apperceptive/associative congenital prosopagnosia proposed advances on previously proposed models of prosopagnosia in several aspects:

- Qualitative vs. quantitative shift: Acquired prosopagnosia has been simulated in artificial neural networks by a decreased connectivity which lead to a quantitative decrease of performance [65], [66]. However, in the case of CP several qualitative shifts have been documented (e.g. gaze behavior [35], [36], no inversion effect [15]). Our model proposes that a qualitative shift to serial, featural processing in CP can emerge as the result of quantitative differences in the extent to which distributed information can be encoded holistically.
- Spatial and temporal integration: The model proposed directly relates a deficit in spatial integration of information extraction (holistic processing) to increases in the time spent inspecting a stimulus, i.e. compensatory temporal integration.
- Gaze behavior: Observations that CPs show different fixation patterns [35], [36], have previously been interpreted as the source of the deficit [36]. However, this raises the question why CPs would learn such a defunct gaze behavior in the first place. Our model provides an alternative explanation of dispersed gaze as the result of compensatory processing due to an inability of holistic encoding.

### Diagnosis of Congenital Prosopagnosia

In this study, diagnosis of CP was based on a semi-structured interview including diagnostic criteria such as a reported uncertainty in face recognition, prolonged recognition times surpassing socially accepted time spans, the development of compensatory strategies, anecdotal stories, and familial recurrence. Relying on a structured but subjective assessment of real life difficulties instead of a more controlled assessment of face recognition abilities under experimental settings, has benefits as well as caveats. Roughly speaking, our method of diagnosis increases the validity with respect to actual clinical symptoms but suffers from a decrease in objectivity due to the impossibility of a perfect standardization of diagnostic interviews. To further explain our view, let us consider two possible constellations: A person shows all symptoms of prosopagnosia as described in this paper, but has a normal test score (within 1 SD). Then we would still consider him prosopagnosic because the test does not prove that in real life situations the person will recognize faces correctly and within the socially accepted time. With respect to the results presented here, the inclusion of such clinically positive cases could potentially lead to a decrease in observed differences in face recognition performance between CP and control group. Thus, our estimate of CP deficits has to be considered a conservative estimate of CP deficits possibly underestimating the true magnitude. Any a posteriori exclusion of clinically positive CPs with normal face recognition skills in formal tests would only lead to an increase in the significance of the reported group differences in face recognition tests.

### Cognitive Heterogeneity

The cognitive heterogeneity in congenital prosopagnosia (cf. [24]) raises the question whether there are identifiable subgroups of CP, comparable to those found in acquired prosopagnosia [6]. In acquired prosopagnosia (AP) a mature, fully functional face recognition system is disturbed by an external event, unrelated to the system's past performance. Irrespective of the exact processes underlying functional specialization of cortical regions in the neural system of face recognition, this specialization presumably leads to an alignment between cortical location and functional process [67]–[70]. Damage inflicted to a specific region can therefore lead to restricted deficits, conditional on the interconnectedness and interdependence of the distributed processing [71]. However, in contrast to the acquired form, individuals with congenital prosopagnosia never evolve a functional face recognition system in the first place and their deficit has to be interpreted as an endpoint of a developmental trajectory [72], a mature but dysfunctional system. Thus, even if there is a single initial cause to CP, it would not be surprising to see a stronger heterogeneity in CP compared to a homogeneous group of AP participants, i.e. with the same lesions, based solely on differences in development, e.g. learning of different evasive and compensatory strategies. Since the strategies adopted vary greatly between individual CPs [16], [20], this complicates a categorization of the intrinsic heterogeneity in CP based on a small number of behavioral tests. Thus, in future studies it seems essential to integrate behavioural as well as neurophysiological/-anatomical variability with computational models of CP based on a general theory of visual information processing.

## Materials and Methods

The experiments were conducted at different times and locations. Experiments with CP participants took place at the Institut für Humangenetik, Westfälische-Wilhelms-Universität, Münster, experiments with control participants took place at the Max Planck Institute for Mathematics in the Sciences, Leipzig. Experiments 1–4 were conducted at the end of 2006, experiments 5 and 6 a year later at the end of 2007.

### Participants

In total we tested 16 CP participants and 36 age matched controls. In each experiment for each CP participant we included up to two age and mostly gender matched control participants. Participation of controls varied across experiments (see below) leading to a total number of control participants in excess of 32.

Participants age (at first testing, i.e. end of 2006) varied between 20 and 68 years for the CPs (mean: 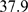, sd: 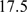) and for the controls (mean: 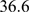, sd: 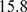). All participants were of caucasian origin.

Except for one CP participant (MB) all CP and control participants reported normal or corrected to normal vision. MB has a strabismus convergens, on which she was operated on three times during childhood. However, she still reported on perceiving diplopic images and difficulties with stereopsy.

#### Ethics Statement

All CP and control participants provided written informed consent before participation. The study was approved by the ethical committee of the University of Muenster, Germany, protocol No 3XKenn2.

#### Participants with Congenital Prosopagnosia

All of the 16 CP participants were diagnosed using a semi-structured interview (see below), which includes questions on everyday-problems with face and object recognition, mental imagery and avoidance strategies.

CP participants - and accordingly control participants - fall into two different age groups: one consisting of 8 younger CP participants, most of them students, aged between 21 and 26 years, the other consisting of 8 older CP participants, aged between 41 and 68.

- The younger group (born after 1980) consisted of five students of medicine, which were detected by a screening study which was conducted by means of a questionnaire (see [19] for a detailed description). Students who reported suspicious behavioral deficits were then invited for the semi-structured interview. In addition, three of the younger participants established contact after having been informed about prosopagnosia via public media or personal contact.
- The older group (age 40 or older) is composed only of people who initiated contact themselves.

See Table 1 for a short overview of CP participants.

**Table 1:** Description of CP participants.

Initials	Contact	Age	Gender
HE	Self-reported	68	F
SE	Self-reported	64	F
EB	Self-reported	57	F
HG	Self-reported	53	M
HB	Self-reported	50	M
MB	Self-reported	48	F
MR	Self-reported	48	F
RK	Self-reported	41	M
JM	Self-reported	26	M
JF	Screening	23	M
HS	Screening	22	M
VK	Screening	21	M
FP	Self-reported	21	F
MG	Screening	21	F
HW	Self-reported	21	F
LL	Screening	21	F

Age is with respect to November 2006 (first series of experiments).

Three CP participants (JM, HW, LL) only participated in experiments 1–4. For one CP participant (MB) online estimation of the 80%-correct presentation time in experiment 1 failed due to technical problems. We therefore excluded MB and both matched controls from all analysis of experiments 1 and 3 which involved limited presentation times.

Most CP participants had intact basic-level object recognition abilities as assessed by a total of seven subtests chosen from the Birmingham Object Recognition Battery [48] - Tests 6,7,10 easy and hard, and 13 - and the Visual Object and Space Perception Battery [49] - Tests 2,4, and 6. Ten CP participants scored in the normal range in all tests. Only one (HW) had difficulties across several object recognition tests (BORB 10A hard, score of 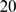 compared to 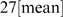 and 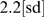, with 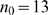; VOSP 2, score of 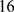 compared to 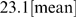 and 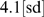; VOSP 4, score of 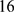 compared to 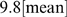 and 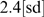; both VOSP with 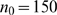). Three older participants (HE, SE, HB) had difficulties in recognizing object silhouettes in VOSP 2 (scores of 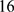, 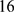, and 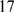 compared to 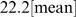 and 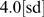). One older participant (HG) had difficulties with VOSP 4 (score of 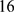 compared to 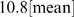 and 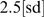). Two (EB, HB) had difficulties with the easy but not the hard condition of BORB subtest 10 (BORB 10B easy, scores of 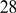 and 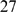 compared to 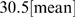 and 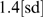, with 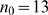). Thus, in all cases including performance was always very close to or at the cutoff level. Furthermore the number of positive findings only deviates slightly from expected values based on percentile cutoff values.

#### Control Participants

In experiments 1–4 a total of 32 control participants (two per CP participant) were selected to match the age of CP participants and also gender in most cases. In experiments 5 and 6 a total of 24 age and mostly gender matched controls participated. Younger control participants were mostly students, similar to CP participants, while the older control participants showed a similar variety in profession as the older CP participants. In total we tested and analyzed the data of 36 controls. 20 controls are included in all experiments, 12 only in experiments 1–4, four only in experiments 5 and 6.

#### Diagnostic Interview

Diagnosis of prosopagnosia was made by a semi- structured interview of about 90 minutes [16]–[20], [50]. In order to be diagnosed as having CP participants had to meet the following criteria:

- Uncertainty in face recognition: Not recognizing familiar people unexpectedly or in crowed places, confusing unknown persons with familiar persons. Only anecdotal mentioning of not recognizing people is not taken as a positive criterion.
- Prolonged recognition time for faces (in terms of a socially accepted span of time).
- Development of compensatory strategies as sign of a longstanding frequent problem. Strategies can include either adaptive behaviour (identification by voice, gait, clothing …) or avoidance behaviour (cancel meetings, looking absent-minded,…).
- Surprising anecdotal stories (problems in following actors in a movie)

In addition, a family history of at least one affected first degree relative renders an hereditary origin of the difficulties more likely, thereby increasing the probability of congenital prosopagnosia -including hereditary prosopagnosia.

### Experiments 1 to 4

#### Stimuli

The face stimuli were obtained from the publicly available Face Database of the MPI for Biological Cybernetics (see [42] for details on the database creation) containing snapshots and 3D-head models obtained by 3D-scans of caucasian people, living in Tübingen, Germany. The database contains snapshots of 3D-scans of 200 heads (without hair), taken at seven rotations (frontal view and 3 rotations in each direction of 30°, 60° and 90°). Snapshots were used as distractor stimuli. Target face stimuli were generated using the four individual full head models in the Face Database (two male and two female heads). Snapshots of the full head models under the same rotations (30°, 60° and 90°) were generated using Blender free open source 3D content creation suite (http://www.blender.org, open-source). All snapshots are 8-bit color images of 256×256 pixels and were presented as colored images in the experiments.

The shoe stimuli were obtained as snapshots (256×177 pixels, 8-bit color) of different sneakers obtained from http://nikeid.nike.com. A total of 53 distractor shoes and 4 target shoes were used, all under the available three different rotation conditions (oblique, side and top view) and presented in color.

A randomly chosen subset of 16 distractor objects was used during the learning blocks, and the remaining distractor items were used in the testing blocks. Each distractor stimulus was exclusively used either during learning or testing but could appear multiple times throughout the experiments. This split ensured that participants learned to recognize targets and not distractors. All matched controls had exactly the same experimental setup (choice of distractors objects during learning and testing as well as presentation order) as their respective CP participants.

#### Design

Experiments 1 to 4 all started with a simultaneous presentation of the same 4 target images in frontal view (faces) or oblique view (shoes) for unlimited duration, which was then followed by a feedback training round. Each training round consisted of 16 trials with frontal/oblique view presentation of images, 8 of which showed the targets (2 times each) and 8 showed a distractor (1 time each). The participant had to respond by clicking the left mouse button for a target and the right mouse button for a distractor (two alternative forced choice - 2-AFC). In the training feedback (correct/false) was given after the response. Presentation and feedback training were repeated until participants made at most a single error during the 16 training trials. Selection of distractor stimuli (8 out of the preselected 16) and the presentation order of target and distractor images was randomized prior to each training block, but exactly the same for a CP participant and his/her two matched controls.

After successful completion of the training the test started (see Figure 1 A for a schematic depiction of the experimental design). In the midst of each test another round of presentation and feedback training was administered. As during training, participants were asked whether the stimulus presented was a target (left mouse button) or a distractor (right mouse button).

The first experiments (1 and 2) for each stimulus class (faces or shoes) tested recognition of the frontal view of target images under varying presentation times (PT) in a two alternative forced choice task. The presentation times were chosen according to the accelerated stochastic approximation method [51], [52] with a threshold at 80% correct, see below. The algorithm increased presentation times, whenever a mistake was made and decreased presentation time after a correct answer. Trials were grouped into blocks of 8 such that in each block every target appeared exactly once. Presentation order of target and distractor image was randomized, but equal for a CP participant and his/her two matched controls.

In experiments 3 and 4 for each stimulus class, we tested recognition of targets in the frontal view and under rotations in a two alternative forced choice task (for faces: 3 in-depth rotations of 30°, 60° and 90°in each direction; for shoes:side view and top view). For faces the test contained two testing blocks of 56 images each (7 rotation conditions with 4 targets and 4 distractors each); for shoes it contained four testing blocks of 24 images each (3 rotation conditions with 4 targets and 4 distractors each). Presentation order of target and distractor stimuli, and rotation angles was randomized in each block, but equal for a CP participant and his/her two matched controls. The presentation time was set to a fixed value chosen for each CP participant individually as an estimate of the time that he/she would need to give correct answers 90% of the time if tested with frontal face images (see below for details of the estimation process). This presentation time was also used for both matched controls of the CP participant.

### Experiments 5 and 6

#### Stimuli

All stimuli were generated with the assistance of the Recognition and Categorization Group in the Department Bülthoff at the Max-Planck-Institute for Biological Cybernetics, Tübingen, Germany. Face images were obtained by rendering from a total of 96 full 3D head models obtained by 3D-scans of caucasian people, living in Tübingen, Germany. The acquisition of 3D-scans and the generation of the head models is described in [42].

For each test we selected 48 individuals, 24 male and 24 female faces. For each individual face we rendered 5 face images, differing in rotation and illumination. One reference image (target stimulus) was taken in frontal view with ambient illumination (rgb = 0.5 0.5 0.5) and an additional white illumination (rgb = 0.7, 0.7, 0.7) from a direction in front, above and to the right of the face (horizontal rotation = 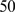
°, vertical rotation = 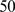
°). Four test images were taken under slight horizontal and vertical rotations of 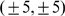 degrees. In all test images the position of the white illumination source was changed to come from in front, below and to the left of the face (horizontal rotation = 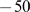
°, vertical rotation = 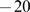
°).

All reference images were standardized to the same rectangular area (i.e. width×height) of the facial image at roughly 25000 square pixels. Resulting images had widths between 122 and 138, and heights between 181 and 204 pixels. Size variations (standard deviation divided by mean) in width and height were slightly smaller compared to human anthropometric measurements [53]. Resulting images were placed upon a black background such that each face was in the center of a 140×210 pixels image.

Test images were not standardized as the standardization in reference images already discounted all size differences with respect to a scaling of the whole image.

#### Experimental Design

In experiment 5, a target stimulus was first presented for a short duration of 50, 150, 450 or 750 ms, and after a blank of 500 ms, the target stimulus had to be recognized among a total of four face stimuli (see Figure 3 A). The response was indicated by pressing one of the four arrow keys corresponding to the position of the stimuli (left, right, up or down). During the recognition the four face stimuli were presented until a response was made. The position of the target stimulus in the test display was randomized. All combinations of 48 target stimuli and four presentation times were tested exactly once per participant, yielding a total of 192 trials. As each stimulus was used both as target in four trials and as distractor in 12 trials, the order of the trials in which each stimulus was presented as target or as distractor was counterbalanced across presentation times to exclude influences of familiarity on the estimation of presentation time effects.

In experiment 6, four target stimuli were initially learned over three rounds of unlimited presentation and subsequent feedback training, and afterwards the influence of variable presentation time on recognition performance was tested (see Figure 3 D).

In each training trial either one of the four target stimuli or one of four different distractors was shown and participants had to indicate, whether the presented stimulus was one of the four target stimuli. Each training round included 8 training trials, where each target and each distractor were presented exactly once in a randomized order.

In each test trial participants had to indicate whether the presented stimulus was one of the four target stimuli learned in the previous training. Each test round consisted of 32 test trials: 16 target presentations and 16 distractor presentations. In every round, each target was presented once for each duration of either 50, 150, 450, or 750 ms. The four distractor stimuli used in the training and an additional four new distractor stimuli were displayed two times each. In these 16 distractor presentations, each of the four presentation durations was chosen four times, but no distractor stimulus was shown at the same duration twice.

The full cycle of presentation, feedback training and test was repeated 12 times such that each of the 48 face stimuli was exactly once among the target stimuli, once among the distractors already present during the training and once among the additional distractors introduced in the test round. Presentation order was counterbalanced across stimuli, groups and presentation time.

In both tests - but not in the feedback training in experiment 6 - test and reference images differed in size, rotation and illumination (see above).

The presentation times of 50, 150, 450 or 750 ms were chosen to separate between different types and numbers of saccadic eye-movements (express-saccades, saccades). As the experiment should be applicable to participants of different age, we relied on studies explicitly addressing age differences in the times needed to perform saccadic eye-movements [54]. However, it is possible that true saccade times found in face processing deviate [28].

### Presentation

In experiments 1–4 images were displayed on either an IIYAMA Vision Master Pro514 monitor (22′, at 200 Hz) or an IIYAMA Vision Master 506 (21′ at 170 Hz) (random assignment, identical for each CP and his/her matched controls) both running at a 800×600 resolution with a screen area of approximately 400 mm×300 mm. Participants were initially seated at a distance of 1m and stimuli covered on average 140 pixels×210 pixels, i.e. 70 mm×110 mm, equal to a visual angle of 4.0°horizontally and 6.3°vertically at a distance of 1 m.

In experiments 5 and 6 presentation was always on the IIYAMA Vision Master Pro514 monitor (22′, at 200 Hz) used previously with a resolution of 800×600 and images subtended 130 pixels×190 pixels, i.e. 65 mm×85 mm or 3.5°×4.3°at the initial seating distance of 1 m.

All experiments were run using the open-source flashdot experimental psychophysics presentation software ([55], available at http://www.flashdot.info), which allows a high temporal precision of the presentation. Presentation duration was actually measured in frames, durations are reported in ms for convenience and to enable comparisons between results obtained at difference monitors with different frame rates. To convert between frames and ms, we simply multiplied the number of frames by the inverse frame rate, which can deviate from the actual presentation times for very small durations [56].

### Statistical Analysis

If not noted otherwise, all data analysis and statistical testing was done in the statistical programming language R [57].

#### Estimation of Presentation Times

In experiments 1 and 2, accelerated stochastic approximation [51] was used as an online estimation method to obtain a presentation time at which participants would make correct responses in 80% of the trials (PT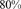). A fraction of 80% correct answers was chosen to avoid ceiling and/or bottom effects and achieve efficient sampling [58], [59]. Given a sequence of at least two answers 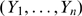, where 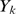 is 1 for a correct answer and 0 for an incorrect answer and an initial presentation time (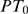), presentation times in the 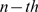 trial (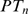) were adjusted according to
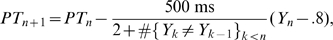
(1)where the denominator contains a counter of the number of shifts in the answer from correct to incorrect or vice-versa.

As the participants completed the tasks on two different monitors with frame rates of 170 Hz and 200 Hz respectively (see below) the actual presentation time sequences had step sizes of 5 ms and 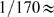
6 ms. The mean of the presentation times of the last 16 trials was taken as an estimate of PT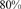.

The data obtained in experiments 1 (or 2 resp.), i.e. all pairs 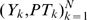, was used to estimate a presentation time for each participant at which he/she would achieve 90% correct answers (PT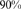). In a pilot study using an 80% correct time we observed bottom effects in performance on rotated images, and thus increased the threshold to 90%.

The software package psignifit (http://www.bootstrap-software.org/psignifit/) was used to fit a modified logistic regression model as an estimate of participants psychometric function relating the response to the presentation time [58]:
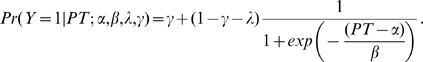
(2)Here, we used as guess rate 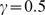, an upper bound on the lapse rate 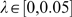 and restricted the shape of the logistic regression function by requiring a positive intercept 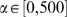 and slope 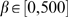. The PT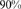 obtained by inverting the fitted logistic regression model in equation (2) to the data gathered in experiment 1 (or 2 resp.) was then used as a presentation time in experiment 3 (or 4 resp.). Each CP participant as well as the two matched controls were shown images at the PT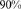 estimated for the CP participant.

In an a-posteriori check, the estimates obtained for the 90%-correct presentation time appeared appropriate, as for presentations of frontal images using these PT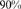 estimates the median CP recognition of frontal views (93% for faces, 95% for shoes) was only slightly above the target rate (see Figure 1 E).

### Generalized Linear Mixed Models

To assess differences in the influence of experimental variables between the control and the CP group, generalized linear mixed models (GLMMs) were used (see e.g. [47] for an introduction to GLMMs). In this section, we only provide a short outline of the statistical methods used. A more detailed explanation is given in Appendix S1.

First, a nullmodel was fitted that always included fixed effects to incorporate influences of age and all experimental variables (e.g. presentation time) as well as random effects allowing for individual variation in the mean and also in the influence of experimental variables. Based on this nullmodel, alternative, nested models were constructed by subsequently adding group differences in the influence of fixed effects, i.e. firstly a mean difference between the groups (main effect), secondly an interaction of group and experimental variables (first-order effects), (see below for details). Comparison of nested models was based on differences in the log-likelihood of the models, i.e. a likelihood ratio test (LR-test).

In addition to model based comparisons, we calculated residuals for each participant using a modified cross-validation approach. The obtained residuals measure the deviation of CP participants performance from the expected performance of a hypothetical control participant with identical individual characteristics (e.g. age). First, parameters of the nullmodel were estimated using only control participants' observations, and the resulting parameter estimates were used to calculate residuals for the observations of CP participants. Second, for control participants, an approach similar to a leave-one out cross-validation was applied: For each control participant, parameters of the nullmodel were estimated using the observations of all control participants except the latter one, and the estimates were then used to calculate residuals for this control participant. Residuals were averaged across observations into a single number for each individual participant. For group comparisons of these average residuals the non-parametric Wilcoxon rank sum test, also referred to as Mann-Whitney test, was used since CP participants' residuals were in most cases not Gaussian-distributed.

Fitting of generalized linear mixed models (GLMMs) was done using the R packages lme4 [60] and MCMCglmm [61]. The algorithms used in lme4 as well as the model based comparisons conducted here, are described by the main contributor to the lme4 package in more detail in [62]. To test for significant differences likelihood ratio tests were performed where we assumed a 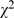 distribution of the test statistics with degrees of freedom equal to the difference in the number of parameters. In testing significance of fixed effects in mixed models, the 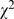 approximation tends to produce to 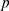-values that are too small [62]. Hence, if the selected model included interaction effects, the model was again fit with MCMCglmm to obtain Bayesian maximum posterior estimates (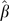) )and highest posterior density intervals with 95% support (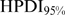) for parameter estimates of interaction effects [63]. As prior distributions for the Bayesian model fitting we used a multivariate normal distribution with zero mean and a diagonal covariance matrix with large variances (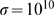) for fixed effects and an inverse Wishart distribution with degrees of freedom equal to one and the inverse scale equal to the unconditional variance of the response variable.

To analyze group differences in reaction times in experiments 1 and 2, a nullmodel including age and training block number as fixed effects was fitted with individual random effects on mean reaction time as well as on the influence of block number. The inverse of the reaction time was taken as the dependent variable to improve model fit (see below). Comparison of group differences in the presentation time needed for 80% correct recognition (PT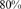) was based on a linear model. The nullmodel only included age as a fixed effect and PT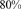 was log-transformed. To assess, whether for faces the group differences found in reaction times can be explained by group differences in presentation times, again nullmodels were constructed for both dependent variables but this time without a prior transformation, and the respective residuals, as well as the difference in residuals was calculated.

Differences in the influence of rotation and presentation time on control and CP participants' performance in experiments 3 and 4 were tested based on binomial GLMM nullmodels with logit-link including with age, rotation angle (nominal scale according to the absolute values, i.e. 0, 30°, 60°, and 90°) and presentation time (log-transformed and mean-centered) as fixed effects and participant identity as random effect. Interactions were tested both for rotation and presentation time combined (full interaction model), as well as specifically for group differences in the influence of presentation time (PT interaction model) to account for a priori known differences in the experimental setup (see above).

Analysis of recognition rates in experiments 5 and 6 was based on a binomial GLMM nullmodel with logit-link including age and the logarithm of presentation time (during training or during recognition resp.) as fixed effects and participants identity as random effect. Reaction times were transformed by taking the inverse (see below) and then analysed using a LMM nullmodel with the same fixed and random effects.

### Analysis of Reaction Times

Feedback training was the same in experiments 1 and 3 (faces) as well as in experiments 2 and 4 (shoes). To increase the sample size, observations were thus pooled across experiments into two data sets (faces and shoes) and the analysis of reaction times during feedback training was then performed conjointly. The results of this conjoint analysis are reported below as results for experiment 1 (faces) and 2 (shoes). As some participants had initial difficulties in understanding the task, we excluded reaction times measured during the very first feedback training from the analysis in order to preclude an influence of individual difficulties in getting accustomed to the experimental setup. In addition, we restricted analysis to trials where a correct answer was given (5349 out of 5424 observations). A fixed cutpoint for observations of reaction time was used dismissing observations with reaction times above 2000 ms and below 500 ms to reduce the number of outliers [64]. A total of 5080 observations of reaction time was analyzed, 2908 observations for face stimuli and 2172 observations for shoe stimuli.

In experiment 5, only reaction times between 500 ms and 8000 ms (corresponding to the 0.01% and 95% quantiles) were included in the analysis.

In experiment 6, only reaction times between 500 ms and 4000 ms (corresponding to the 0.05% and 96% quantiles) were included in the analysis. Note that participants could respond only after the end of the stimulus presentation.

Prior to any analysis using model based comparisons, reaction times were transformed by taking the inverse to improve applicability of linear models [64].

## Supporting Information

Appendix S1 Detailed description of the statistical methods used: Generalized Linear Mixed Models and model based normalization of test scores.
(0.14 MB PDF)
