# Supplementary material for: The Early Time Course of Compensatory Face Processing in Congenital Prosopagnosia
Source: PLoS One. 2010 Jul 21;5(7):e11482. doi: 10.1371/journal.pone.0011482 (PMC2908115; doi:10.1371/journal.pone.0011482)
Supplement: Appendix S1 — Detailed description of the statistical methods used: Generalized Linear Mixed Models and model based normalization of test scores. (0.14 MB PDF) [file pone.0011482.s001.pdf]

The Early Time Course of Compensatory Face Processing in Congenital Prosopagnosia - Supporting Information

Appendix A - Generalized Linear Mixed Models

A linear model relates an outcome random variable Y to a set of predictor random variables $X = (X_1, \ldots, X_J)$ via a linear map: $Y = \sum_{j=1}^{J} \beta_j X_j$. A Generalized Linear Mixed Model (GLMM) is a generalization of linear (fixed effect) models in two ways (see [1] for a review):

- linearity is generalized by the introduction of a link function g:

$$E[Y] = g^{-1}(\sum_{j=1}^{J} \beta_j X_j)$$

- random effects allow to incorporate information on correlated observations, which arise e.g. due to repeated measurements taken from the same individual in different conditions

Let $\nu := \sum_{j=1}^{J} \beta_j X_j$ be the linear predictor. It is assumed that there is a functional relationship, specified by the link function g, between this linear predictor and the expected observed outcome, i.e. $\nu = g(E[Y])$. For random variables Y which have a distribution from the exponential family, g is mostly chosen such that $\nu = g(E[Y]) = \theta$, where θ is the canonical (or location) parameter of the distribution. In this case, the link-function is called the canonical link-function [2]. Examples for canonical link-functions are the identity function, $g(x) = x$, for normal distributions and the logit-function, $g(x) := \log(\frac{x}{1-x})$, for Binomial distributions.

Usually, the link-function is fixed *a priori* and estimation of the model parameters is limited to the coefficients β_j and selection and/or transformations of the predictor variables X_j. Introducing a differentiation between fixed and random effects, the general form of a GLMM can be written as follows

$$\nu(X) := \beta_0 + \sum_{j=1}^{J} \beta_j X_j + \sum_{k=1}^{K} \gamma_k Z_k, \qquad (1)$$

where β_0 is the intercept, β_j are the fixed effect coefficients for the observed X_j and γ_k are the random effects for the observed Z_k.

Nested Families of Generalized Linear Mixed Models

Model based comparisons can be used to study whether the influence of fixed effects on the outcome differs between groups. First, construct a nested family of models. Starting from a nullmodel

$$\nu_0 := \beta_0 + \sum_{j=1}^{J} \beta_j X_j + \sum_{k=1}^{K} \gamma_k Z_k,$$

more complicated models are constructed by allowing for interaction effects between predictor variables X_j and a grouping variable C. For example, a main-effect (or 0th-order) model can be defined by adding group specific intercept terms β_c for all groups c. Similarly, a 1st-order effect

models can be defined by including interactions with all predictor variables X_j, i.e. by adding terms $\beta_{c,j}\, X_i$. More general, a kth-order model can be defined by including interactions with all interaction terms of length k ($\beta_{c,j_1,\ldots,j_k}$).
To test whether the influence of predictor variables differs significantly between groups we used likelihood-ratio tests. For two nested models, a null model M_0 and an alternative model M_1, with respective log-likelihoods $l_0 < l_1$, $2(l_1 - l_0)$ was calculated as a test statistic.

In general, it is assumed that this test statistic follows χ^2 distribution with degrees of freedom equal to the difference in the number of parameters. However, in most cases, the assumption of a χ^2 distribution is only an approximation and tends to give to small p-values [2]. This shortcoming can be addressed by applying resampling methods (e.g. parametric boostrap) to estimate p-values or Bayesian statistics.

Appendix B - Model Based Normalization of Test Scores

Investigating whether an individual's performance in behavioural tests deviates fundamentally from that of a control population is one of the main aims of a quantitative diagnostic assessment. To conduct a quantitative diagnosis one has to arrange for a matching control population, derive a sufficient statistical description, and decide whether an individual's performance deviates significantly. A commonly used method is to select for each individual a control population that is matched in terms of possible contributing factors (age, gender, education,...), calculate mean and standard deviation of the matched controls, and derive an abnormality score based on an appropriate test statistic [3]. However, restricting the comparisons to matched controls decreases the number of samples in the control group and often introduces a somewhat arbitrary discretization of continuous variables (e.g. age) into intervals (e.g. age-bands). By applying regression methods to model the influence of contributing factors on test performance one can establish continuous norms [4].

We propose to use generalized linear mixed models to extend simple linear regression methods in accounting for differences in possible contributing factors and deriving continuous norms. The main extension is the possibility to transform outcome variables that don't follow a normal distribution, e.g. Bernoulli or exponential distributed random variables into residuals that are approximately normal distributed. This transformation ensures applicability of standard test statistics [5].

First, a nullmodel is fitted to the observed control data. This initial process of fitting or constructing a nullmodel establishes which of the possible factors actually contributes to control performance. Only those are included as predictor variables in the construction of a continuous norm. Second, for each control individual i with observed outcome y_i and predictors (contributing factors) x_i residuals are calculated as the difference in actual performance y_i and expected performance under an individualized nullmodel $\hat{y}_{-i}(x_i)$. The individualized nullmodel is obtained by estimating the parameter values of the nullmodel based on all control observations except those of individual i. Third, for each new individual j, residuals can be calculated similarly, this time based on difference between observed performance y_j and expected performance under the nullmodel using all control observations $\hat{y}(x_j)$.

The additional step of calculating controls' residuals based on individualized nullmodels

reduces the risk of fitting model parameters too closely to the data, thereby modeling the idiosyncrasies of each individuals' performance and underestimating the variability in control performance (cf. to a leave-one-out crossvalidation, see [6]).

References

1. Tuerlinckx F, Rijmen F, Verbeke G, Boeck PD (2006) Statistical inference in generalized linear mixed models: a review. The British journal of mathematical and statistical psychology 59: 225–55.

2. Faraway JJ (2006) Extending the linear model with r: generalized linear, mixed effects and nonparametric regression models. Chapman & Hall, FL : 301.

3. Crawford J, Garthwaite P (2002) Investigation of the single case in neuropsychology: confidence limits on the abnormality of test scores and test score differences. Neuropsychologia 40: 1196–1208.

4. Crawford J, Garthwaite P (2006) Comparing patients' predicted test scores from a regression equation with their obtained scores: A significance test and point estimate of abnormality with accompanying confidence limits. Neuropsychology 20: 259–271.

5. Crawford J, Garthwaite P, Azzalini A, Howell D, Laws K (2006) Testing for a deficit in single-case studies: Effects of departures from normality. Neuropsychologia 44: 666–677.

6. Efron B, Tibshirani R (1993) An introduction to the bootstrap. Chapman & Hall : 436.
